# A Developing Symbiosis: Enabling Cross-Talk Between Ecologists and Microbiome Scientists

**DOI:** 10.3389/fmicb.2019.00292

**Published:** 2019-02-20

**Authors:** Laura Tipton, John L. Darcy, Nicole A. Hynson

**Affiliations:** ^1^Department of Botany, University of Hawai’i at Mānoa, Honolulu, HI, United States; ^2^Pacific Biosciences Research Center, University of Hawai’i at Mānoa, Honolulu, HI, United States

**Keywords:** ecology, microbiome, succession, symbiosis, diversity

## Abstract

Like all interactions, the success of cross-discipline collaborations relies on effective communication. Ecology offers theoretical frameworks and lexicons to study microbiomes. Yet some of the terms and concepts borrowed from ecology are being used discordantly by microbiome studies from their traditional definitions. Here we define some of the ecological terms and concepts as they are used in ecology and the study of microbiomes. Where applicable, we have provided the historical context of the terms, highlighted examples from microbiome studies, and considered the research methods involved. We divided these concepts into four sections: Biomes, Diversity, Symbiosis, and Succession. Biomes encompass the interactions within the biotic and abiotic features of an environment. This extends to the term “microbiome,” derived from “biome,” and includes an environment and all the microbes within it. Diversity encompasses patterns of species richness, abundance, and biogeography, all of which are important to understanding the distribution of microbiomes. Symbiosis emphasizes the relationships between organisms within a community. Symbioses are often misunderstood to be synonymous with mutualism. We discard that implication, in favor of a broader, more historically accurate definition which spans the continuum from parasitism to mutualism. Succession includes classical succession, alternative stable states, community assembly frameworks, and *r/K*-selection. Our hope is that as microbiome researchers continue to apply ecological terms, and as ecologists continue to gain interest in microbiomes, each will do so in a way that enables cross-talk between them. We recommend initiating these collaborations by using a common lexicon, from which new concepts can emerge.

## Background

In order for two entities to communicate effectively, they must start by speaking the same language. This applies equally to organisms engaging in symbioses and to scientists engaging in cross-discipline collaborations. Scientists who study host-associated and other microbiota typically draw on many different disciplines to do so, including microbiology, computer science, molecular biology, statistics, and medicine. In addition to these fields, ecology provides theoretical frameworks for the study of microbiomes and can be at least as influential as these other disciplines. How to apply ecological theory to microbiomes has been discussed previously; for key examples, see Costello et al. (2012), who promote applying community assembly theory to human microbiomes Koskella et al. (2017), who highlight some of the challenges of applying current ecological theories of community assembly, complexity, and dynamics to microbiomes, and Gonzalez et al. (2011), who suggest how ecological principles and models can be used as predictive models for personalized medicine.

We propose that synergy between microbiome and ecological research will be strongest and most enduring when researchers speak the same language. To encourage continued cross-talk and fruitful collaborations between disciplines, and to evolve the field of ecology by incorporating microbes, we present a brief overview of some of the ecological terms and concepts that are most pertinent to the study of microbiomes. Some of these terms and concepts are used in disparate ways among many ecologists and microbiome researchers. To harmonize this discord among fields we have provided the historical contexts for how these terms evolved and at times, diverged among sub-disciplines, and suggest how to move forward. In Table 1 we present an alphabetical listing of these terms and concepts along with our recommended definitions, some of which originate from ecology, and others from microbiome studies. Similar to recent advances in quantum physics owed to the development of new methodologies for subatomic exploration, the rapidly expanding field of microbiome research is leading to revisions of classical ecological theory, some of which we highlight here.

**Table 1:** Quick reference of terms and definitions.

Term	Recommended definition
Alpha Diversity	Diversity, or variety, within a sample or group. Some metrics emphasize richness or evenness, and may or may not be weighted by abundance of the species
Alternative Stable States	The assembly of a community dictated by the timing of the disturbance, the available species pool, biotic and abiotic interactions
Bacteriome, Mycobiome, Virome	All genetic material from bacteria, fungi, or viruses, respectively, present in an environment
Beta Diversity	Diversity, or dissimilarity, across samples or groups. Like alpha diversity, some metrics may be weighted by species abundance
Biome	Biotic and abiotic components that define an ecosystem, specifically physiography and latitude
Biogeography	Distribution of organisms
Climax Community	A final stable state of community composition
Commensalism	Type of symbiosis where one partner benefits without any measurable effect on the other
Community Ecology	Diversity and interactions of organisms within a given area
Dysbiosis	Unbalancing of microbial community composition or function of the microbiome within a host
Ecological Networks	Representations of the pairwise biotic interactions of an ecosystem, interactions may be observed or inferred
Evenness	Component of alpha diversity that measures if all species are present in approximately the same abundance
Filter - Dispersal	Selective process whereby a species must be able to arrive at the ecosystem to be part of the community
Filter - Environmental	Selective process whereby a species must be able to survive in the environment to be part of the community
Filter - Interaction	Selective process whereby a species must be able to survive with or outcompete existing species to be a part of the community
Holobiont	Assemblage of participants in a symbiosis
Hologenome	Combined genomes of all parts of the holobiont
Microbiome	Microorganisms and abiotic conditions that define an environment
Metagenome	All genomic material present in an environment
Mutualism	Type of symbiosis where both partners benefit
Neutral Processes	Community assembly processes where all species are assumed to be functionally equivalent or equally likely to occur
Niche Processes	Community assembly processes where the resource availability determines species composition
Parasitism	Type of symbiosis where one partner benefits at the expense of the other
Phylosymbiosis	The mirroring of the phylogenetic distance between hosts by the diversity of their associated microbial communities
Resilience	A property of stable states, characteristics of the community that act to retain the current community composition
Resistance	A property of stable states, tendency for a community to remain in its current state
Richness	Component of alpha diversity measuring the number of species present in a sample or group
*r/K* selection	Combination of life history traits associated with many offspring that are poor competitors (*r*-selection) or few offspring that are strong competitors (*K*-selection)
Species Abundance Distribution (SAD)	Model of the abundance and rareness of all species within an ecosystem
Species Turnover	Transitions in community composition due to appearances and disappearances
Succession	Process of change in the species composition of a community post disturbance and over time
Symbiosis	Interaction among species
Syntrophy	Type of mutualistic symbiosis where all partners depend on each other metabolically

*The terms and concepts discussed in this review are presented with suggested definitions for use in both ecology and microbiome studies.*

Ecology is the study of the interactions of organisms with each other and their environments (Real and Brown, 1991), and as such, can contribute significantly to the study of microbiomes. Specifically, biogeography, the geographic distributions of organisms, and community ecology, the diversity of, and interactions among “species,” have been the focus of many of many early microbiome studies, even if the fields were not recognized by name. We have identified four additional facets of ecology that provide relevant frameworks and lexicons for the study of microbiomes: Biomes, Diversity, Symbioses, and Succession.

## Biomes – Micro and Macro

**FIGURE 1:**
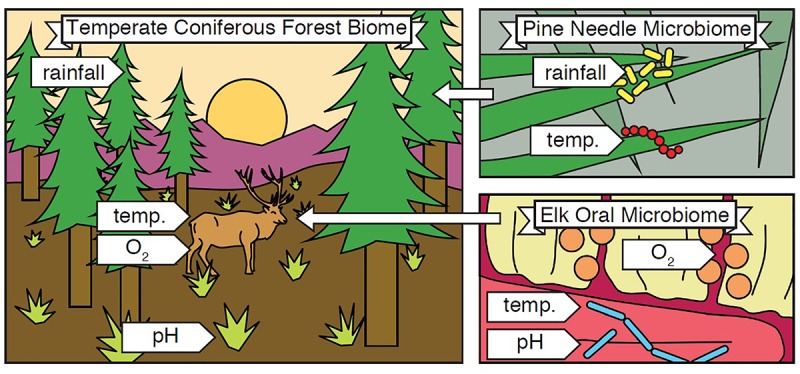
Biome and Microbiome. A biome encompasses all organisms and abiotic conditions of an ecosystem, and microbiomes are nested within it. In the illustrated example, a temperate coniferous forest biome **(left)** contains organisms including pine trees and elk, as well as abiotic conditions such as rainfall, temperature, oxygen availability, and soil pH. The biome also contains countless microbial species, and each microbiome within it contains a subset of these. For example, the pine needle microbiome **(top right)** contains microorganisms and abiotic conditions found in or around pine needles, and the elk oral microbiome **(bottom right)** contains microorganisms and abiotic conditions found in the mouths of elk. In this way, “pine needle microbiome” refers to the organisms and abiotic conditions within and around pine needles in general. The example depicted is only one sample, just as one human gut is just a sample of the broader human gut microbiome.

The term biome was originally defined by Clements and Shelford in their 1939 book “Bio-Ecology” to define the plant-animal communities of an environment (Clements and Shelford, 1939). These two authors were operating within the paradigm of the environment-as-a-complex-organism analogy (Law, 1992), and biome was their way of encompassing all plants, animals, and the local environment in which they live and interact (Figure 1). Along geographic longitudes^1^ biomes tend to be similar, changing as one moves along a latitudinal gradient, making them larger than a single habitat.

Despite the longstanding definition of biome in ecology, in the short history of microbiome studies, there has been controversy over how to define the word. Jonathan Eisen provides a brief and personal history in his blog post, “What does the term microbiome mean? And where did it come from? A bit of a surprise” (Eisen, 2015). It boils down to one question: does the word derive from the suffix “-ome,” meaning all of, like “genome,” or is it a portmanteau of “microbe” and “biome”? Eisen, history, and key reviews (Ursell et al., 2012; Marchesi and Ravel, 2015) agree that the definition of microbiome should be based on the latter. Thus, the microbiome encompasses all of the microbes and the environment, both biotic and abiotic factors, in which they live (Figure 1 inset).

Yet, some still use microbiome to indicate the collection of genes from all of the microbes present (Koskella et al., 2017), which is more properly referred to as the metagenome. Adding to this confusion is the fact that bacteriome, mycobiome, and virome represent the genetic material of the bacteria, fungi, and viruses present in the ecosystem, respectively. As macro-ecological biomes are not subdivided into taxa-specific biomes such as the animal biome and the plant biome, neither should microbiomes be subdivided into the bacterial biome, fungal biome, viral biome, and so on. Because many studies are limited to the genetic material from a certain subset of the microbiome, in some cases these taxonomically explicit terms (“bacteriome”) do have utility. For example, a study that uses a marker-gene approach (e.g., 16S rRNA gene) to survey a microbiome may be limited in scope to bacteria and archaea, although eukaryotes and viruses were present in that microbiome as well. In this case, using a word like “bacteriome” communicates the scope of the study.

Although emphasis has been placed on bacterial members of microbiomes, these studies have described both the abiotic conditions and biotic (bacterial) inhabitants that define particular microbiomes. For key examples, see publications out of the Human Microbiome Project (The Human Microbiome Project Consortium, 2012), Earth Microbiome Project (Thompson et al., 2017), and MetaHIT (Arumugam et al., 2011). With previous limitations to a holistic approach to studying microbiomes disappearing (such as cost, technology, and computational resources), going forward, we encourage microbiome researchers to continue to describe “microbial biomes,” and to incorporate other inhabitants including fungi, viruses, archaea, and protists. Considering the spatial and temporal scales relevant to the ecological study of microbiomes and what factors (biotic or abiotic) lead to the assembly of specific microbiomes also deserves additional attention. As pointed out by Koskella et al. (2017), the spatial and temporal scales that influence microbiomes are likely to be much smaller and shorter for microbiota than macrobiota due to their size and often high rates of reproduction.

## Diversity

The most basic definition of diversity is variation within a group or alpha-diversity. However, ecological diversity can be measured in many ways, and use of the term “diversity” itself is not uniform within the field. For example, species richness is the simplest type of alpha-diversity, and it is a count of the number of species observed. A sample containing many species is said to have higher richness or alpha-diversity than a sample containing fewer species (Whittaker, 1972). Microbiome scientists familiar with software packages like QIIME, Phyloseq, and Mothur will note that all of these tools use the term “alpha-diversity” in the same sense as the basic definition, variation within a group (Schloss et al., 2009; Caporaso et al., 2010; McMurdie and Holmes, 2013).

However, the definition of the word “diversity” in ecology is contentious, variable, and... diverse. Many ecologists argue that diversity must include richness as described above, but also evenness, which is a measure of how uniformly distributed species abundances are within a sample. To illustrate, consider two samples A and B, both of which have richness of 3. In Sample A, one species comprises 99% of the observations, but in sample B, each of the 3 species is equally abundant. Thus, sample A has low evenness, since its distribution of species abundances is very skewed, while sample B has high evenness because its distribution of species abundances is even. Even though the samples have the same richness, it is clear that sample A has lower diversity than sample B when evenness is considered.

While this is a strong argument that diversity must include evenness (for metrics that include evenness, see Shannon diversity and phylogenetic entropy; Rosenzweig, 1995; Allen et al., 2009), it is not always the case in the ecological and microbiome vocabulary. The term alpha-diversity includes several metrics that are not abundance-weighted and therefore do not include evenness. Phylogenetic diversity accounts for the shared evolutionary history among species within a sample, but it is not inherently abundance weighted (Faith, 1992). Yet the word “diversity” is still present in the metric’s name, and under the “alpha-diversity” umbrella as well. While there have been proposals that would remedy this discrepancy, including one by Tuomisto (2010), the word “diversity” is frequently used to include both weighted and un-weighted metrics, within ecology, microbiome science, and colloquially.

In ecology, “diversity” also often implies the difference between two or more samples. This definition is a large departure from “variation within a group,” because it is explicitly between or among groups instead of within. A pair of samples that are very compositionally dissimilar from each other are said to have high beta-diversity, and a pair of samples that are identical are said to have zero beta-diversity. This term, along with alpha-diversity, was popularized by Whittaker (1972), who described it as “the extent of species replacement or biotic change along environmental gradients.”. The term “compositional dissimilarity” is sometimes used in lieu of “beta-diversity,” and often the name of the beta-diversity metric is directly invoked in microbiome studies (e.g., “Bray-Curtis dissimilarity”). Just like alpha-diversity metrics, beta-diversity metrics may or may not include differences in richness, evenness, or phylogenetic relationships among species. For example, Jaccard distance is a beta-diversity metric that includes only presence-absence of species in its calculation, while Bray-Curtis distance includes relative abundances of those species as well. Beta-diversity metrics may account for phylogenetic relationships among species as well, as is the case with UniFrac (Lozupone and Knight, 2005; Lozupone et al., 2011).

Although diversity is currently measured similarly across microscopic and macroscopic life, not all broad-scale diversity patterns documented for plants and animals hold true for microbes. For example, one of the most well-known and well-conserved biogeographic patterns among macro-organisms is that species diversity increases from the poles to the equator, known as the “latitudinal gradient of diversity” (Pianka, 1966; Willig et al., 2003; Hillebrand, 2004). However, studies of planktonic bacteria near the surface of the open ocean have been shown to have less consistent patterns with peak diversity either at temperate latitudes (Ladau et al., 2013; Milici et al., 2016) or near the equator (Fuhrman et al., 2008). Even more extreme, ectomycorrhizal fungi, a group of fungi that form intimate symbioses with plant roots, have the highest diversity in the Holarctic, or high northern latitudes rather than the tropics (Tedersoo et al., 2010). Several explanations for these differing or reverse latitudinal gradients of diversity among microbes have been proposed, including higher rates of dispersal and lower rates of extinction (Fenchel and Finlay, 2004; Fuhrman et al., 2008), but the reason for this observation may be as simple as under-sampling of microbes (Tedersoo et al., 2010).

The shape of species abundance distributions (SADs), which models the abundance of all species present in an ecosystem and whose shape is related to evenness, is another well-conserved pattern of diversity among macro-organisms that diverges in microbes. The SAD of macro-organism communities most often fits as a log-series distribution, while lognormal distributions provide a better fit for microbial community SADs (Shoemaker et al., 2017). This difference is likely driven by different ecological processes acting on the communities, including growth rates and dispersal limitations. These distributions can be used predictively for conservation and other applications (reviewed in Matthews and Whittaker, 2014) so understanding how SADs differ across domains of life will provide important and critical information for species conservation.

## Symbiosis

**FIGURE 2:**
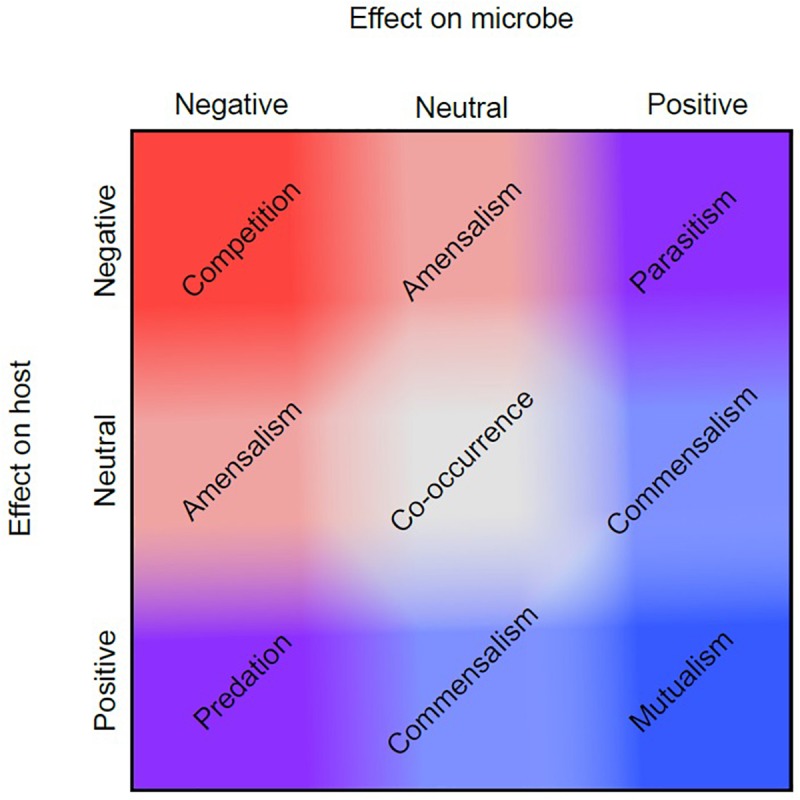
Symbiotic relationship continuum. Symbiotic relationships encompass multiple dimensions of effect, represented here on two axes. If a symbiosis has a positive (blue) effect for a microbe, and a negative (red) effect for the host, this is known as parasitism (top right corner). However, each type of symbiosis shown does not occupy a discrete factorial combination of positive and negative effects. Instead, some symbioses may have more positive or negative effects for a symbiont or host than others, and these may shift depending upon their environmental context as shown in the figure by the gradation of red and blue values between the two axes. For example, two different symbioses may both be mutualistic (mutually positive, bottom right), but one of those relationships may stray slightly more toward commensalism. This figure is adapted from Bronstein (1994).

Historically, among biologists there has been disagreement on the proper use and definition of the term symbiosis, which is derived from the Greek “syn” meaning together and “bios” meaning life (Symbiosis| Origin and Meaning of Symbiosis by Online Etymology Dictionary, 2017). While Heinrich Anton de Bary is credited with popularizing the term in 1879, it was first used in 1877 by Albert Bernhard Frank in reference to the coexistence of different species (Smith and Read, 2008; Oulhen et al., 2016). Both Frank and de Bary used the term “symbiosis” to refer to all types of interactions between species ranging from parasitism – where one partner benefits at the expense of the other(s), to commensalism – where one partner benefits without any measurable effect to the other(s), to mutualism – where all partners benefit (Figure 2) (Sapp, 2004).

In the century following de Bary and Frank, symbiosis became synonymous with mutualism among most biologists, while few continued to use the term more broadly. It was not until the late 20th century that the initially intended, and more broad definition of symbiosis became (re)popularized spurred by the work of Starr (1975) who proposed eight criteria for symbiotic interactions, Lewis (1985) who further modified these criteria to include competitive, amensalistic, agonistic, and neutral interactions, and more recently Bronstein (1994) and Thompson (2005) who highlighted the conditionality of the outcome of symbiotic interactions. Microbial interactions continue to expand our understanding of symbiosis through examples such as syntrophy, where species rely on each other metabolically (Morris B.E.L. et al., 2013). This extreme mutualism is sometimes simplified as “cross-feeding” but can also take the form of individuals “cheating” mutualisms through adaptive gene loss, as proposed in the “Black Queen Hypothesis” (Morris et al., 2012). Future studies of microbiomes will continue to inform and refine our understanding of the breadth of biotic interactions, and may lead to reconsiderations of what constitutes a symbiosis.

For example, in light of our modern recognition of the importance of symbiotic microbial communities, the term holobiont was coined in 1991 to describe the assemblage of participants in a symbiosis (Margulis and Fester, 1991). One of the more well studied symbiotic assemblages is the coral holobiont which includes the coral animal, the dinoflagellates of the genus *Symbiodinium* that live within the coral’s gastrodermis layers, and the microbiota that live both in and on the coral animal (Bourne et al., 2016). These coral associated microbiota include bacteria (Raina et al., 2009), archaea (Siboni et al., 2008), fungi (Amend et al., 2012), and other eukaryotes that can be distinguished based on coral animal species and tissue specificity. While still an area of active research, it is hypothesized that members of a holobiont, such as the coral holobiont, contribute to the fitness of the assemblage by incurring such traits as stress tolerance (Bordenstein and Theis, 2015). Application of the holobiont concept and its associated hologenome to other organisms, including humans, is more controversial, with skeptics pointing out that other evolutionary explanations, including host selectivity or natural selection acting upon microbiota, may provide an equally simple reason for the resulting assemblages (Moran and Sloan, 2015; Johnson and Foster, 2018).

Currently symbiosis is considered a central aspect of biology (Sapp, 2004) and is taught in ecology classes under the broader definition of “living together,” which can be refined by adjectives including “mutualistic,” “commensal,” or “parasitic” (Martin and Schwab, 2012). However, the use of the term among microbiome researchers has retained the connotation of mutualism. This is likely due to the parallel evolution of the term “dysbiosis” in the late 19th century among medical professionals whose research focused on animal gut microflora (Hooks and O’Malley, 2017). Dysbiosis is broadly defined as the “unbalancing” or change in community composition and function, often associated with disease, but not necessarily with a specific pathogen (Tamboli et al., 2004). Dysbiosis has been referred to as the opposite of symbiosis, eubiosis, homeostasis, or normobiosis (Hooks and O’Malley, 2017). This profusion of terms has led to incongruences among the medical and ecological literature, which can be addressed by using the terms in their broadest senses in combination with appropriate modifiers for increased specificity, e.g., “mutualistic symbiosis” and “disease-associated dysbiosis.”

Symbiotic relationships among interacting species have been studied in numerous ways to determine their placement along the continuum of mutualism to parasitism. Among macro-organisms these relationships are often studied by direct observation, such as witnessing a behavior in the field as in the case acacia trees and their ant mutualists (Young et al., 1996). Symbiotic relationships among microbes have also been studied using direct experimentation, such as the co-culturing of parasitic TM7x oral bacteria with its host bacteria *Actinomyces odontolyticus* (He et al., 2015) and the carbon source phenotype array testing of the fungus *Fusarium keratoplasticum* with its endohyphal mutualist bacterium *Chitinophaga* sp. (Shaffer et al., 2017). Microbial symbioses provide many promising study systems for direct experimentation to increase our understanding of the evolution and ecology of symbiosis. For example, experiments are being conducted on the well-characterized system of wasps in the genus *Nasonia*, their obligate intracellular bacteria *Wolbachia* spp. and *Arsenophonus nasoniae*, which are reproductive parasites, and the other bacteria that live primarily within the wasp gut (Dittmer et al., 2016). The vertical transmission from parent to offspring of *A. nasoniae* and its amenability to genetic manipulation makes this symbiosis especially attractive for direct experimentation. For mammalian hosts, germ-free and gnotobiotic (colonized with a known, simpler than wildtype microbial community) rodents are used to study host-microbe and microbe-microbe interactions *in vivo* (Martín et al., 2016).

Working with a well-described microbial community in a well-studied host allows for the exploration of host effects, including genetics, diet, and stress to name a few, on the microbiome. As host effects on microbiota and vise-versa become better understood, the influence of the environment on microbiota and, in-turn their hosts are the next frontiers to be explored. Environmental effects have long been studied by ecologists using common garden experiments, which can test for local adaptation or plasticity by exposing multiple ecotypes to standardized environmental conditions and measuring fitness (de Villemereuil et al., 2016). Common garden experiments can also be extended to interactions with potentially confounding variables including microbes, such as when Quigley and co-authors grew a macroalga in a common garden to examine the effect of spatial heterogeneity on the macroalgal microbial community structure (Quigley et al., 2018). Common garden, among other manipulative methods have long histories of well-designed experiments that can be adapted from ecology to help microbiome researchers test and understand the environmental effects on the microbiome.

Biotic interactions can also be studied through non-observational or computational methods, such as bipartite interaction networks among plants and pollinators (Olesen and Jordano, 2002) or by indirect interaction network models among microbiota (Friedman and Alm, 2012; Kurtz et al., 2015). Interaction networks among microbes have the advantage of being able to uncover relationships between unculturable microbes that would not be detectable by direct observation. One caveat of the network-based methods for determining the presence of symbiosis is that these inferred interactions may represent only the extreme positive and extreme negative ends of the mutualism to parasitism spectrum, particularly if these interactions are inferred based on a predetermined threshold that discounts mildly correlated species pairs. These network methods are not limited to a single microbial Kingdom and have been used successfully to examine bacteria-fungi interactions (Tipton et al., 2018). For example, in the human oral microbiome, *Streptococcus* sp. bacteria and *Candida albicans* yeast come together to form corncob structures in the plaque biofilm (Zijnge et al., 2010), and soil-dwelling *Streptomyces hygroscopicus* bacteria cause *Aspergillus nidulans* fungi to produce secondary metabolites only when in direct contact (Schroeckh et al., 2009). Additional bacteria-fungi interactions have been more comprehensively reviewed by Tarkka and Deveau (2016). Unfortunately, pairwise interaction networks will not predict if symbioses occur between more than two species, such as fungal farming ants that use antibiotic producing bacteria of the order Actinomycetales to protect their gardens of the *Leucoagaricus gongylophorus* fungus from pathogens (Barke et al., 2010). The ability to predict such higher-order interactions and multipartite interactions within networks is an area of ongoing investigation in graph theory that is beginning to be applied to ecological networks (Estrada et al., 2008; Layeghifard et al., 2017) and should also be considered in microbiome studies.

Interaction networks, or ecological networks, represent an area of active research which is poised to illuminate snapshots of interactions among microbes and their hosts. However, the plasticity of ecological networks in relationship to the abiotic environment and over time remain unintegrated into current models. Thus, carefully designed experiments that test how symbiotic relationships and the ecological networks in which they are imbedded may change depending upon environmental context and across temporal scales are desperately needed. Microbiome studies of well understood model hosts exposed to variable environments provide promising study systems with which to address this. For example, the lifespan of *Drosophila* has been shown to decrease two-fold in flies whose gut microbiome has been experimentally eliminated through heat treatment (Yamada et al., 2015). The relationship between microbiomes and hosts may always be biased toward the perspective of the health of the host, as reflected by the term dysbiosis, but has the potential to become more nuanced as our understanding of the holobiont intra- and inter-trophic microbial interactions increases and more states of health and disease are studied.

## Succession

**FIGURE 3:**
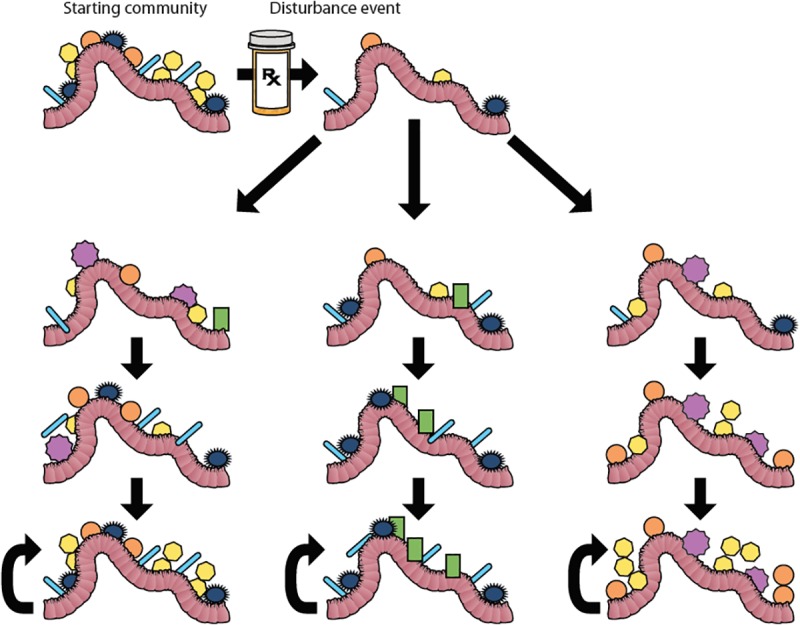
Succession and Alternative Stable States. Microbial communities within the gut can be disrupted by a disturbance event like antibiotic treatment. In this figure, the starting community represents the microbial community in the gut before antibiotic treatment. Antibiotic treatment changes the composition of the gut bacteria. After the treatment, the community re-assembles. The left column represents succession culminating in a climax community that mirrors the original starting community. Under historical definitions of the word “succession” (*sensu* Clements, 1916), this re-assembly to a deterministic climax community would be the only trajectory called succession. However, under modern usage of the term, succession includes the possibility of alternative stable states (Beisner et al., 2003), which are shown in the middle and right columns. In these cases, different stable communities may be reached depending on the order of species arrival into the disturbed environment. Each alternative stable state has resilience, which is symbolized by the curved arrows.

Ecological succession is the process of change in the species composition of a community over time after a disturbance such as a fire or landslide. Succession is divided into primary succession of newly created habitats and secondary succession of disturbed communities (Figure 3). For many years succession was thought of as an ordered sequence of communities building to a climax community, or ideal community composition dictated by the environmental factors within the biome (Law, 1992), but this idea has fallen out of favor along with biomes-as-complex-organisms analogy that it was a part of Real and Brown (1991). This paradigm shifted in the mid-late 20th century in part because, unlike an organism where a juvenile develops into in adult, communities do not always develop into the same climax state following disturbances. The idea of a single homeostatic community composition for a microbiome (Zaneveld et al., 2017) is reminiscent of the climax community concept, so much so that cystic fibrosis researchers termed the community that dominates the lung microbiome during periods of disease stability the “climax community” in their climax-attack model (Conrad et al., 2013), despite acknowledging that there may be multiple climax communities. Much like macro-biomes, microbiomes can exist in alternative stable states (Figure 3). Alternative stable states develop depending on the order of species arrival to the new environment. Once established, communities have positive feedbacks that builds resilience, characteristics that act to retain the current community composition. In order to shift between states, communities must overcome resistance, the tendency for a community to remain in its current state, as can happen to the gut microbiome during a course of antibiotics (Costello et al., 2012). One of the goals of ecologists who study succession is to model and predict future communities based on pre-disturbance composition, source pools for recolonization post-disturbance, and type of disturbance. Applying these approaches to the medical study of microbiomes would be a boon to the pre- and pro-biotic industries.

Studies of succession are typically interested in species turnover, or transitions in community composition due to the disappearance of some species and the appearance of others. Disappearances may come from local extinction or emigration out of the area, and appearances may come from immigration into the area or speciation. Due to the relatively slow rate of reproductive isolation leading to speciation, within ecological timeframes species turnover due to speciation is often low to the point of negligible among macrobes (Volkov et al., 2003; Hubbell, 2005). However, species turnover can be much higher among microbes, particularly bacteria which may speciate rapidly due to shorter generation times and horizontal gene transfer (Shapiro et al., 2016). Rapid turnover, due to both speciation and immigration, creates a challenge when applying traditional theories and models to microbial communities, not only in the study of succession, but also in studies of other ecological concepts including species interactions, dispersal, heritability, and community assembly (Koskella et al., 2017). One way to combat this challenge is to evaluate microbial communities at the strain level, rather than the species-equivalent (OTU) level (Truong et al., 2017). Some even go so far as to say that OTUs, particularly at the 97% similarity threshold, should be considered obsolete and should be replaced with oligotypes (Eren et al., 2013) or amplicon sequence variants (ASVs) (Callahan et al., 2017).

Another concept closely associated with succession is that of *r/K* selection. This theory comes from the 1970’s and includes suites of traits associated with either *r*- or *K*-life history strategies (MacArthur and Wilson, 2001). Often, but not always linked to early succession, *r*-selection traits include fast growth rates, producing many offspring with low survival rates, often with small body size and early sexual maturity. In contrast, *K*-selection traits are often linked with later stages of succession gradually replacing the *r*-strategists. *K*-selection traits include producing fewer offspring that are superior competitors and often include large body-size and long life. Within a decade of its introduction, *r/K* selection theory was considered to be an over-simplified view of life history trait tradeoffs (Stearns, 1977). However, recently, the terms have been picked up by microbiome researchers, largely for their associations with growth rates under differing resource availability (Saleem, 2015), i.e., that *r*-strategists are opportunistic and thrive during times of surplus while *K*-strategists are more specialized to thrive during times of deficit. Vadstein et al. (2018) even went so far as to recommend *K-*strategists as the desirable type of bacterial community members for aquaculture instead of recommending a bacterial community composition or management strategy. Yet *r*- and *K*-selection continue to be an oversimplification or shorthand for a suite of traits rather than a definitive characterization of a species’ life history.

Ecological theories on community assembly processes abound and provide promising frameworks to examine microbial communities (Hubbell, 2005). Commonly, models of community assembly processes take one of two forms: neutral, wherein all species are assumed to be functionally equivalent or equally likely to occur, or niche, wherein community composition is, at least partially, a function of environmental conditions. Despite being modeled separately, niche and neutral *processes* themselves are not necessarily mutually exclusive and both can work in tandem to arrive at the same community (Leibold and McPeek, 2006; Dumbrell et al., 2010; Chase, 2014). Another common community assembly framework is the “filter” model, whereby members of a regional species pool must pass through a series of filters, or selective processes, in order to survive as members of a local community (Poff, 1997). These filters include: a dispersal filter, a species must be able to arrive at the ecosystem, an abiotic or environmental filter, they must be able to survive in the environment to which they arrive, and finally a biotic or interaction filter, they must be able to either coexist with or out-compete the organisms already present in the community. These community assembly frameworks are being used occasionally in microbiome studies; for example, the inability of neutral process models to predict the composition of the lung microbiome based on the oral microbiome was used as evidence for a lung-specific (niche) microbiome (Morris A. et al., 2013), and environmental filters including both the stream location and host micro-environment (gills vs. carapace) were shown to shape the microbiomes of crayfish (Skelton et al., 2017). The crayfish example, among numerous others, illustrates how, in the context of the assembly of host associated microbiomes, the host should be considered as an additional biotic filter which often strongly influences microbial community composition (Ley et al., 2008; Reveillaud et al., 2014; Martínez-García et al., 2015).

## Moving Forward, Together

As ecological and microbiome studies continue to intersect, there must be room for the growth and development of both fields based the outcomes of these collaborations. For example, phylosymbiosis, the phenomenon where the similarity of the host-associated microbial communities mirrors the phylogeny of the hosts (Brooks et al., 2016), is one such new term coined in 2013 (Brucker and Bordenstein, 2013). While shared evolutionary history may not be the only factor leading to similar microbial communities among hosts, the hypothesis of phylosymbiosis provides an example of the emerging recognition of the interconnection and interdependency of micro- and macro-organism evolution.

Additionally, the virome, and its interactions with the rest of the microbiome and hosts represents an emerging area of research where we predict ecologists and microbiome researchers will further develop fertile collaborations. Research is already being conducted on host-associated viromes and their impact on the inter-kingdom interactions between host and microbes (Leigh et al., 2018). Ecological models and concepts drawn from our vast knowledge of predator-prey interactions are primed to be explored in phage–bacteria–host systems.

## Conclusion

Microbiome research is inherently trans-disciplinary making it an ideal emerging field within which we can break down perceived silos and open doors to new concepts that will need to be defined and explored through the lens of historically separate fields. As more studies of microbiomes go beyond listing the community members to describing their function and exploring their broad patterns, more ecological concepts will be borrowed, necessitating this cross-talk between ecologists and those who study microbiomes. Here we have outlined some of the terms and concepts borrowed from ecology that are already used in the study of microbiomes in the hopes that an agreed upon lexicon will ease these types of collaborations.

## Author Contributions

LT and NH conceived of and wrote the initial manuscript. JD revised the manuscript and designed the figures. All authors approved the manuscript.

## Conflict of Interest Statement

The authors declare that the research was conducted in the absence of any commercial or financial relationships that could be construed as a potential conflict of interest.

## Footnotes

1 It is worth noting that “longitudinal” in human microbiome studies seems to originate from the medical and statistical fields, where it describes a study with a temporal component (i.e., samples collected over time) whereas in ecological studies, longitudinal can also mean having to do with the longitude (and latitude) lines that encircle the earth.
